# Variation in serum urate levels in the absence of gout and urate lowering therapy

**DOI:** 10.1186/s41927-021-00202-6

**Published:** 2021-09-08

**Authors:** Andrew Shaffer, Elizabeth Rahn, Kenneth Saag, Amy Mudano, Angelo Gaffo

**Affiliations:** 1 Division of Rheumatology, University of Utah, 30 N 1900 E, SOM4B200, Salt Lake City, UT 84132 USA; 2 Division of Rheumatology, University of Alabama at Birmingham, SHEL 306 1530 3rd Ave S, Birmingham, AL 35294 USA

## Abstract

**Background:**

Previous studies have noted significant variation in serum urate (sUA) levels, and it is unknown how this influences the accuracy of hyperuricemia classification based on single data points. Despite this known variability, hyperuricemic patients are often used as a control group in gout studies. Our objective was to determine the accuracy of hyperuricemia classifications based on single data points versus multiple data points given the degree of variability observed with serial measurements of sUA.

**Methods:**

Data was analyzed from a cross-over clinical trial of urate-lowering therapy in young adults without a gout diagnosis. In the control phase, sUA levels used for this analysis were collected at 2–4 week intervals. Mean coefficient of variation for sUA was determined, as were rates of conversion between normouricemia (sUA ≤6.8 mg/dL) and hyperuricemia (sUA > 6.8 mg/dL).

**Results:**

Mean study participant (*n* = 85) age was 27.8 ± 7.0 years, with 39% female participants and 41% African-American participants. Mean sUA coefficient of variation was 8.5% ± 4.9% (1 to 23%). There was no significant difference in variation between men and women, or between participants initially normouricemic and those who were initially hyperuricemic.

Among those initially normouricemic (*n* = 72), 21% converted to hyperuricemia during at least one subsequent measurement. The subgroup with initial sUA < 6.0 (*n* = 54) was much less likely to have future values in the range of hyperuricemia compared to the group with screening sUA values between 6.0–6.8 (*n* = 18) (7% vs 39%, *p* = 0.0037).

Of the participants initially hyperuricemic (*n* = 13), 46% were later normouricemic during at least one measurement.

**Conclusion:**

Single sUA measurements were unreliable in hyperuricemia classification due to spontaneous variation. Knowing this, if a single measurement must be used in classification, it is worth noting that those with an sUA of < 6.0 mg/dL were less likely to demonstrate future hyperuricemic measurements and this could be considered a safer threshold to rule out intermittent hyperuricemia based on a single measurement point.

**Trial registration:**

Data from parent study ClinicalTrials.gov Identifier: NCT02038179.

## Background

In addition to its well-described role in the pathogenesis of gout, hyperuricemia (defined as a serum urate [sUA] >  6.8 mg/dL) is associated with vascular, cardiac and renal disease [1–9]. This hyperuricemic threshold, or similar ones based on sUA distributions in populations of men and women, are commonly used in epidemiological and clinical research [10, 11].

However, the reliability of a single sUA measurement in defining hyperuricemia is unclear. Previous studies have found significant variability in the measurement of serum urate over time, without known causes for the fluctuation. Suggested reasons for this fluctuation include variations in diet, alcohol intake, body weight, time of day, and hydration status. Hourly [12] and seasonal variation in serum urate levels has also been suggested [13–15].

Many epidemiological studies which have explored the question of whether sUA levels are associated with cardiovascular outcomes have used a single-point threshold (such as 6.8 mg/dL or 7.0 mg/dL) to classify individuals as hyperuricemic [10, 11]. Even when hyperuricemia is not defined, sUA levels are often stratified based on a single measurement [16, 17].

The large number of factors affecting SUA likely account for their propensity to fluctuate. Because of this variability, more research is needed as to the value of a single SUA level in confirming or discarding hyperuricemia. Our study results will have implications for epidemiological and clinical studies in hyperuricemia and gout, which sometimes rely on a single sUA measurement to classify participants. Further evaluations of serum urate variability over a short period of time could demonstrate the utility in performing serial checks prior to classification of patients in both study and clinical situations.

## Methods

Data from the “Serum Urate Reduction to Prevent Hypertension (SURPHER)” study were used. The study protocol has been described in detail [18]. Briefly, SURPHER is a single center, cross-over clinical trial conducted in the city of Birmingham, Alabama (USA) that tested the hypothesis that serum urate reduction with allopurinol could be beneficial in elevated blood pressure in young adults. It was approved by the Institutional Review Board (IRB) at the University of Alabama at Birmingham (UAB – IRB approval number 130408004). Oversight was provided by a Data Safety Monitoring Board. The trial was conducted in accordance with the provisions of the Declaration of Helsinki and the International Conference on Harmonization Good Clinical Practice Guidelines and registered in clinicaltrials.gov (NCT02038179). Written informed consent was obtained from all participants. Key enrollment criteria for the SURPHER trial were 1) The mean of two clinic measurements with systolic blood pressure (SBP) ≥ 120 and < 160 mmHg or diastolic blood pressure (DBP) ≥ 80 and < 100 mmHg; 2) a serum urate ≥5.0 mg/dL for men or ≥ 4.0 mg/dL for women; and 3) age between 18 and 40 years old. Major exclusion criteria included 1) current pharmacological treatment for hypertension (calcium channel blockers were only allowed); 2) prior diagnosis of gout or past use of ULT for gout; 3) > 2 alcoholic drinks per day; 4) glomerular filtration rate (GFR) <  60 mL/min/1.73 m^2^. Full description of enrollment criteria and study procedures have been published previously [18].

Participants were initially selected via a telephone screen, followed by a study visit in which a screening sUA was first measured at an appointment in either morning or afternoon. After a 2-to-4-week run-in period during which participants received a daily dose of placebo to assess adherence, a sUA level was measured a second time prior to any intervention.

Study participants were subsequently randomized to receive oral allopurinol 300 mg daily or placebo for 3 weeks, and a sUA was measured a third time. After a 2-to-4-week washout period, sUA was re-measured prior to crossing over to the other study arm for another 3 weeks. A sUA was then measured a fifth and final time at completion of the study.

The 4-week washout interval before crossover between study arms allowed for full dissipation of the effects of allopurinol, which is converted to oxypurinol and has a half-life of 18–30 h [2]. This dissipation was anticipated to take place over no longer than 2 weeks after the last dose of allopurinol. To confirm this in our study, carry-over effects were examined using a two sample t-test, described in detail in the SURPHER study [18].

Samples for sUA level measurements were processed using two Beckman Coulter instruments. Precision statistics performed on these instruments in 2014 demonstrated an observed coefficient of variation in serum urate of 0.3–1.2%.

Except for the initial screening visit, which occurred throughout the day, serum urates were planned to be drawn during appointments between the hours of 0700 and 1100. Participants were asked to fast during the day prior to the screening visit. The samples were collected at a consistent time within the menstrual cycle of premenopausal females enrolled in the study.

For our secondary analyses of the data initially obtained during the SURPHER trial, we made use of sUA levels collected at screening, pre-intervention, pre-placebo, and post-placebo. Levels collected following intervention were not considered in our analysis. Only the data from the participants that had measurements of all four sUA levels occurring without intervention was used for our analysis. Mean coefficient of variation for sUA was determined and compared across groups (sUA levels at screening, gender) using t-test statistics to determine differences.

The rates of conversion from normouricemia (sUA ≤6.8 mg/dL) to hyperuricemia (sUA > 6.8 mg/dL), and from hyperuricemia to normouricemia were calculated. The rates of conversion to hyperuricemia were then compared across subgroups defined by the sUA level at initial screening, as well as between subgroups with only one initial normouricemic reading (and a second check which was hyperuricemic) versus two initial normouricemic readings. Comparisons between count data in groups were performed using Fisher’s Exact tests, due to small cell size.

## Results

Of the 99 SURPHER study participants enrolled, 85 completed all four sUA measurements that occurred in the absence of allopurinol (during the placebo period). The 14 participants who did not complete all four checks were excluded from further analysis. Time of day data was available for 76% of participants, and showed that 84% of measurements outside of the screening sUA occurred during the targeted time frame of 0700 to 1100. Mean ± standard deviation study participant (*n* = 85) age was 27.8 ± 7.0 years and mean body mass index was 31.1 ± 7.9. 39% of participants were women. 41% of participants were African-American. (Table 1).

**Table 1:** Characteristics of study population

Characteristic	Study population (*n* = 85)	Normouricemic at Screening (*n* = 72)	Hyperuricemic at Screening (*n* = 13)
Age at Enrollment (mean ± SD)	27.8 ± 7.0	27.9 ± 7.1	27.4 ± 7.3
Sex			
Male	52 (61%)	39 (54%)	13 (100%)
Female	33 (39%)	33 (45%)	0 (0%)
Race/Ethnicity			
White	45 (53%)	37 (51%)	8 (62%)
African-American	35 (41%)	32 (45%)	3 (23%)
Other	5 (6%)	3 (4%)	2 (15%)
BMI (mean ± SD)	31.1 ± 7.9	30.8 ± 8.1	32.4 ± 6.6
Screening Serum urate (mean ± SD, mg/dL)	5.8 ± 1.2		
Men	6.4 ± 1.0		
Women	4.9 ± 0.7		

*SD* Standard deviation, *mg/dL* Milligrams per deciliter

The mean coefficient of variation in sUA for all qualifying participants was 8.5% ± 4.9% (1 to 23%). There was no significant difference in the coefficient of variation between men (8.5%) and women (8.6%) (*p* = 0.88), or between subjects who were initially normouricemic (sUA ≤ 6.8 mg/dL) (8.6%) and those who were initially hyperuricemic (sUA >  6.8 mg/dL) (8.0%) (*p* = 0.68) (Table 2).

**Table 2:** Variability of serum urate by initial level and by sex

Serum urate at screening (mg/dL)	Number of participants	Mean coefficient of variation of sUA
< 5.0	22	9.4% ± 6.2%
< 6.0	54	8.6% ± 5.1%
≤ 6.8	72	8.6% ± 4.9%
> 6.8	13	8.0% ± 5.5%
All Men participants	52	8.5% ± 4.8%
All Women participants	33	8.6% ± 5.2%
All participants	85	8.5% ± 4.9%

*mg/dL* Milligrams per deciliter, *sUA* Serum uric acid

**Fig. 1:**
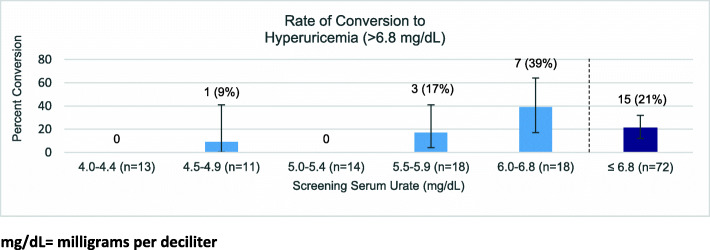
mg/dL = milligrams per deciliter. Of those 13 participants who initially presented with a hyperuricemic sUA, 6 (46.2%) later converted to normouricemia during at least one subsequent measurement without intervention. There was no significant difference in conversion rates between the group with an initial sUA 6.9–7.5 (2/6, 33.3%) and the group with (4/7, 57.1%) (*p* = 0.59) (Fig. 2).

Among the 72 participants with an initial sUA value in the range of normouricemia, 15 (21%) had sUA values in hyperuricemia ranges during at least one subsequent measurement. The subgroup with initial sUA <  6.0 (*n* = 54) was much less likely to have future values in the range of hyperuricemia compared to the group with screening sUA values between 6.0–6.8 (*n* = 18) (7% vs 39%, *p* = 0.0037) (Table 3, Fig. 1).

**Table 3:** Rates of conversion from normouricemia at initial check to hyperuricemia and from below common treatment goals to above common treatment goals at any subsequent serum urate check

Serum urate at screening (mg/dL)	Number of participants	Number of participants converted to > 5		Number of participants converted to > 6		Number of participants converted to hyperuricemia (> 6.8 mg/dL)	
		n	% (95% CI)	n	% (95% CI)	n	% (95% CI)
4.0–4.4	11	2	18% (2–52%)	0	(0%)	0	(0%)
4.5–4.9	11	5	45%(17–77%)	1	9%(0.2–41%)	1	9%(0.2–41%)
5.0–5.4	14	N/A		6	43%(18–71%)	0	(0%)
5.5–5.9	18	N/A		14	78%(52–94%)	3	17%(4–41%)
6.0–6.8	18	N/A		N/A		7	39%(17–64%)
< 5.0	22	7	32%(14–55%)	1	5%(0.1–23%)	1	5%(0.1–23%)
< 6.0	54	N/A		21	39%(26–53%)	4	7%(2–18%)
≤ 6.8	72	N/A		N/A		15	21%(12–32%)

*mg/dL* Milligrams per deciliter

**Fig. 2:**
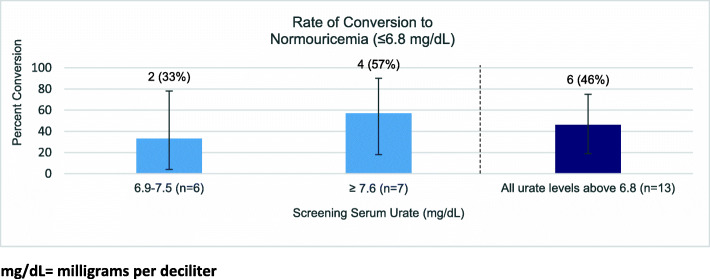
mg/dL = milligrams per deciliter. To determine whether a second normouricemic sUA measurement decreases the rate of subsequent conversion to hyperuricemia, a group with one initial normouricemic sUA (and a second check which was hyperuricemic) was compared to a group with two initial normouricemic sUAs. There was a non-significant trend between the group with only one normouricemic check (*n* = 6) vs. two normouricemic checks (*n* = 66) in conversion to hyperuricemia during the 3rd to 4th sUA checks, with the latter group more likely to remain normouricemic ([3/6 (50%) vs. 9/66 (14%) *p* = 0.0541].

## Discussion

In this study we demonstrated that single-point sUA measurements are unreliable in hyperuricemia classification. About 21% of participants initially normouricemic were found to be hyperuricemic on subsequent checkups. In addition, nearly half of hyperuricemic participants were normouricemic on subsequent checkups. Our findings could influence the way studies using serum urate as an enrollment criteria or outcome are conducted or planned. Our study differed from previous examinations in its calculation of a threshold for screening sUA level more helpful in ruling out future hyperuricemia.

We used 6.8 mg/dL as our threshold for hyperuricemia, as this is related to the physiologic saturation at which urate begins to precipitate. We found a sUA threshold < 6.0 mg/dL, reliable in ruling out subsequent hyperuricemia, as the conversion rate was just over 7%. Our somewhat small number of subjects did not allow us to calculate a comparable threshold value above which persistent hyperuricemia appears to be more likely. A second sUA measurement did not add reliability in excluding future hyperuricemia, as there was no significant difference in the conversion rates between those with one initial value below threshold and those with two values below threshold.

There has been a paucity of published data on the rate of conversion from normouricemia to hyperuricemia and from hyperuricemia to normouricemia after an initial laboratory check in the absence of intervention.

A 2004 study examined the serum urate levels and 24-h urinary uric acid levels monthly for a 12-month period in 12 healthy men on self-selected diets, without medications known to effect urate levels, as well as abstinence from alcohol 7 days prior to measurement of levels. Seven of the twelve subjects (58.3%) had transient hyperuricemia at some point during the study period [19]. A similarly designed study found that 10/12 subjects experienced transient hyperuricemia at some point during the course of 1 year [15]. The Framingham heart study concluded that a higher percentage of the male population had hyperuricemia when considering four biennial determinations rather than a single determination [6].

The mean coefficient of variation for sUA was also examined in these two similar studies [15, 19]. Our mean coefficient of 8.5% was comparable to the findings of the 2004 study *(*9%, with a range of 5–12%) [19], and is less than that found in the second study mentioned (17.5%, with a range of 15–22%) [15].

In addition to variation over months, it has also been demonstrated that uric acid levels fluctuate over the course of a single day [20]. Serum urate levels were significantly higher when measured in the morning than when measured in the afternoon, with a decrease of up to 30% seen in a subgroup of diabetic patients [12]. In contrast to this, other studies have found that the serum urate levels are lowest when measured in the morning [21, 22]. The factors driving diurnal variations in serum urate are not fully understood, but changes in the variation may be impacted by gender, age and diet [22]. Differences in uric acid levels have also been shown to be associated with hypertension with “non-dipping profiles” (absence of significant decrease in blood pressure during sleep), suggesting that there may be interplay between sUA and blood pressure determiners [23–25].

The cause of the variation in sUA found by our study and previous studies is likely multifactorial. Multiple elements are known to affect sUA levels, including diet and medications. Intake of alcohol, purine-rich foods (including seafood and organ meats such as liver), xylitol and fructose have all been associated with increased serum urate. Medications that increase urate include low-dose aspirin, pyrazinamide, cytotoxic chemotherapy, diuretics (particularly loop and thiazide diuretics), immunosuppressants such as cyclosporine and tacrolimus, nicotinic acid, testosterone, levodopa and theophylline [26, 27]. Lead exposure also increases urate and can cause saturnine gout [28]. Disease processes that increase serum urate include renal failure, polycythemia vera, chronic myeloid leukemia and other hematologic malignancies, genetic diseases such as Lesch Nyhan, and any malady that results in acidemia [12]. Alternately, medications that can decrease SUA levels include losartan, amlodipine, fenofibrate and high dose aspirin [27].

Even though our study participants were part of a clinical trial and sUA measurements were collected in a relatively short time frame (12–14 weeks), the observed variability in sUA levels could possibly be a result of subtle factors such as time of day, diet, changes in weight, renal function and undisclosed use of medications. As more data becomes available regarding the degree to which these variables affect sUA, a more standardized protocol for checking sUA may become common practice, such as checking sUA early in the morning, prior to any meals.

This variability in sUA may also influence treatment guidelines for gout in the future. Perhaps in part due to increased recognition of the imprecision of a single spot serum urate level, there is controversy regarding the choice between titration of urate lowering therapy to minimize acute intermittent symptoms and the treat-to-target approach with specified urate goals [29, 30]. However, because our study population excluded individuals with gout, our ability to extrapolate in this regard is limited.

Our study has a number of limitations. The initial screening sUA was not standardized with regards to time of day as the rest of the measurements were, and not all the subsequent measurements occurred during the targeted time frame. This discrepancy could account for some of the measured variability in sUA levels, as urate concentration is highest in the morning [12]. However, in current clinical practice it is not standard of care to draw sUA levels at a specified time of day, and sUA levels measured at different times are often compared to one another to assess response to treatment. In this sense, our analysis may more accurately represent variability in sUA often demonstrated in clinical practice. The diet of the participants also likely had an impact on the sUA variation. We attempted to mitigate this effect by asking participants to fast prior to measurements, but this overall likely remains a factor in urate variation both in our study and in the general population.

The young age of our participants could limit generalizability to older populations. We used the same definition for hyperuricemia in both males and females, due to the physiologic precipitation of urate at this level; we also attempted to control for changes in urate due to hormonal fluctuations by timing our sample collections at similar points in the menstrual cycles of our participants.

The sample size was relatively small, but our study may prompt future studies with larger numbers of participants. Additionally, the cohort examined was from a single center. Further validation from additional study cohorts, or a multi-center analysis is likely warranted.

A total of 13 study participants presented with an initial sUA in the hyperuricemic range, which could make the conclusions obtained from that group imprecise. This small sub-population size could be the reason why a threshold sUA level above which conversion to hyperuricemia is less likely could not be proposed. Additionally, the enrollment criteria of the parent SURPHER study requiring an initial sUA of ≥5.0 mg/dL for men and ≥ 4.0 mg/dL for women does limit our ability to evaluate serum urate variability in these individuals with very low sUA levels [18].

Several of the measurements of sUA took place outside of the planned 2-to-4 week intervals to maintain consistency in relation to the menstrual cycles of the subjects. This could have limited our ability to compare measurements between subjects, but our demonstrated variability in sUA over time remains valid despite this limitation.

The presence of placebo and allopurinol dosing in between analyzed measurements could have affected our results, though we attempted to minimize these effects by confirming dissipation of the effects of allopurinol by examining carry-over effects with a two sample t-test.

There was also exclusion of 14 out of 99 individuals who did not complete all required sUA checks, which could affect our data if the reason for these omissions were associated with factors affecting serum urate fluctuations.

Participants did receive monetary compensation for their participation in the trial, which could have influenced the characteristics of the study population [18].

## Conclusions

Our data is consistent with previous studies which have found significant variation in sUA levels over time, even when controlling for commonly identified influencers of sUA levels. This finding diminishes somewhat the value of a single sUA check, and even a second measurement was not found to add significant reliability in excluding future hyperuricemia in those who are initially normouricemic. Future studies could examine the reliability of sUA checks in those with gout, as well as those on ULT, to determine whether a treat-to-target approach for gout flare suppression may be subject to these same fluctuations in sUA levels.

## Data Availability

The authors are committed to complying with the NIH Public Access Policy for publications (Consolidated Appropriations Act, 2008; Division G, Title II, Section 218 of PL 110–161). An electronic version of our final, peer-reviewed manuscript upon acceptance for publication, will be made publicly available no later than 12 months after the official date of publication: We will likewise comply with NIH policies regarding public access to datasets generated by this project and public sharing policies for Digital Scientific Data, as described in publication “NIH Plan for Increasing Access to Scientific Publications and Digital Scientific Data” (2/2015).

## Abbreviations

DBP: Diastolic blood pressure
GFR: Glomerular filtration rate
IRB: Institutional Review Board
mg/dL: Milligrams per deciliter
SBP: Systolic blood pressure
suA: Serum urate
SURPHER trial: Serum Urate Reduction to Prevent Hypertension trial
UAB: University of Alabama at Birmingham
ULT: Urate lowering therapy
